# Tumor microenvironment as a complex milieu driving cancer progression: a mini review

**DOI:** 10.1007/s12094-024-03697-w

**Published:** 2024-09-28

**Authors:** Zhengrui Li, Jing Li, Xiaolei Bai, Xufeng Huang, Qi Wang

**Affiliations:** 1 Department of Oral and Maxillofacial-Head and Neck Oncology, Shanghai Ninth People’s Hospital, Shanghai Jiao Tong University School of Medicine, Shanghai, China; 2 College of Stomatology, Shanghai Jiao Tong University, Shanghai, China; 3 National Center for Stomatology, Shanghai, China; 4 National Clinical Research Center for Oral Diseases, Shanghai, China; 5 Shanghai Key Laboratory of Stomatology, Shanghai, China; 6 Shanghai Research Institute of Stomatology, Shanghai, China; 7 Shanghai Center of Head and Neck Oncology Clinical and Translational Science, Shanghai, China; 8 Research Unit of Oral and Maxillofacial Regenerative Medicine, Chinese Academy of Medical Sciences, Shanghai, China; 9 Shanghai Stomatological Hospital & School of Stomatology, Fudan University, Shanghai, China; 10 Department of Stomatology, Zhejiang Provincial People’s Hospital, Affiliated People’s Hospital, Hangzhou Medical College, Hangzhou, China; 11 Faculty of Dentistry, University of Debrecen, Debrecen, Hungary; 12 Department of Gastroenterology, Affiliated Hospital of Jiangsu University, Jiangsu University, Zhenjiang, China; 13 Department of Oncology, Ruijin Hospital, Shanghai Jiao Tong University School of Medicine, Shanghai, China; 14 Digestive Disease Institute of Jiangsu University, Affiliated Hospital of Jiangsu University, Jiangsu University, Zhenjiang, China

**Keywords:** Tumor microenvironment, Cancer progression, Therapeutic resistance, Immune modulation, Cellular interactions, Bioactive molecules, Angiogenesis, Heterogeneity

## Abstract

It has been spotlighted that the Tumor Microenvironment (TME) is crucial for comprehending cancer progression and therapeutic resistance. Therefore, this comprehensive review elucidates the intricate architecture of the TME, which encompasses tumor cells, immune components, support cells, and a myriad of bioactive molecules. These constituents collectively foster dynamic interactions that underpin tumor growth, metastasis, and nuanced responses to anticancer therapies. Notably, the TME’s role extends beyond mere physical support, serving as a critical mediator in cancer-cell evolution, immune modulation, and treatment outcomes. Innovations targeting the TME, including strategies focused on the vasculature, immune checkpoints, and T-cell therapies, have forged new pathways for clinical intervention. However, the heterogeneity and complexity of the TME present significant challenges, necessitating deeper exploration of its components and their interplay to enhance therapeutic efficacy. This review underscores the imperative for integrated research strategies that amalgamate insights from tumor biology, immunology, and systems biology. Such an approach aims to refine cancer treatments and improve patient prognoses by exploiting the TME’s complexity.

## Introduction

Cancer research represents a significant public health menace, where relentless endeavors against carcinogenesis have been at the forefront of scientific exploration. This collective effort has culminated in notable progress, fueling an academic research surge as reflected in a spectrum of high-impact scholarly works. Despite advancements in anticancer pharmacotherapeutics and therapeutic modalities, the persistence of drug resistance and recurrent treatment failures [1] continues to pose colossal challenges in the contemporary oncologic landscape [2, 3]. In light of these challenges, the Tumor Microenvironment (TME) has emerged as a focal point of study, positioned as a potential nexus for novel anticancer drug development [4]. Recognition of its key role underscores a paradigm shift in cancer research [5], highlighting the complex interplay between tumor cells and their surrounding milieu as a determinant of therapeutic outcomes.

## The tumor microenvironment: an overview

**Fig. 1:**
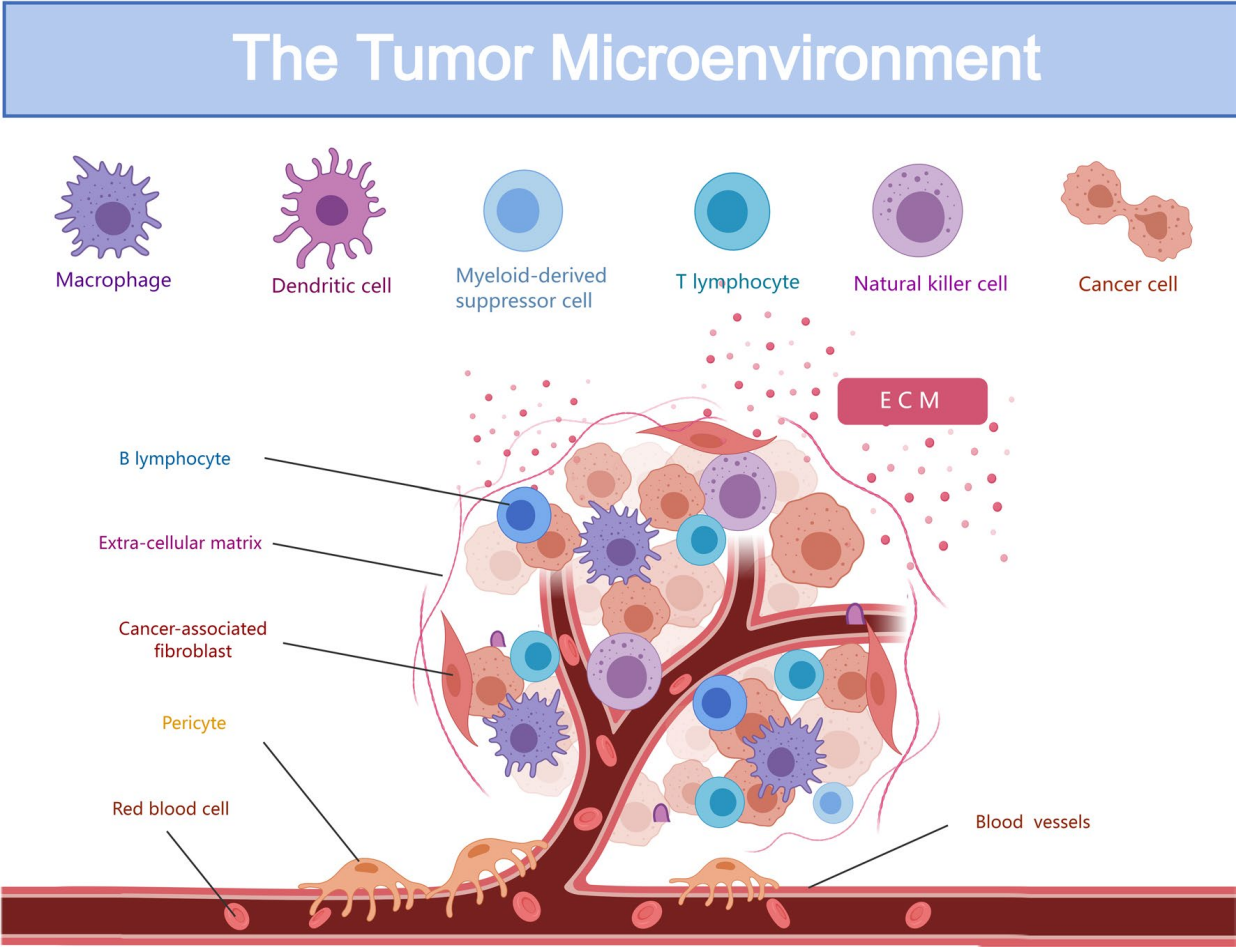
The tumor microenvironment—the intrinsic milieu within which tumor cells thrive and evolve

The TME constitutes the intrinsic milieu for tumor-cell growth and evolution [6, 7]. It comprises tumor cells, immune cells, and supporting cells (such as fibroblasts, stromal cells, and endothelial cells), along with over-secretion of bioactive molecules like cytokines and chemokines [8, 9] (Fig. 1). Major cellular and non-cellular components of the TME are T-cells, B-cells, tumor-associated macrophages (TAMs), natural killer cells, neutrophils, DCs, endothelial cells, cancer associated fibroblasts, adipocytes, stellate cells, extracellular matrix (ECM), and exosomes [10]. In addition, ECM forms a non-cellular component within this environment [11]. Analogously, if we perceive a tumor as a building, the surrounding community and its adjacent structures are a microcosm of the TME.

**Fig. 2:**
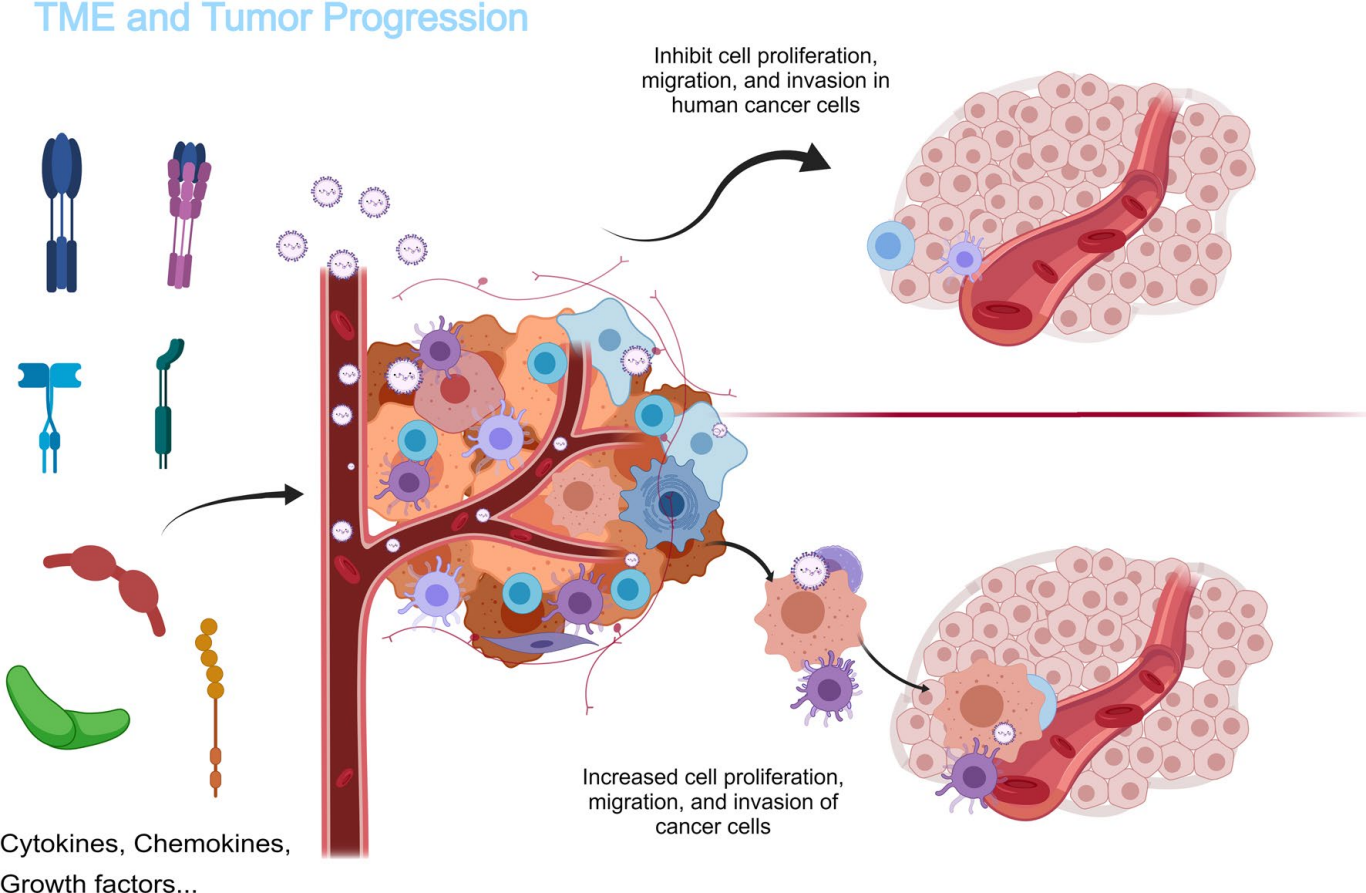
The TME and tumor progression with the erosion and transformation by malignant cells

In the TME, tumor cells manipulate the microenvironment to extract essential resources from surrounding cells [6, 12]. Initially, the microenvironment should act as a bastion against tumor proliferation, preserving the viability of normal cells [13]. However, as the tumor progresses, this once harmonious environment will be eroded (Fig. 2). A deeper academic discussion is required for understanding the multifaceted roles played by different components of the TME. For instance, the ECM is not merely a physical scaffold but actively participates in tumor progression by regulating cell adhesion, migration, and invasion [14]. Similarly, the immune landscape within the TME merits particular attention [15], encompassing a spectrum from anti-tumorigenic immune surveillance to pro-tumorigenic immune suppression, describing a complex interplay that significantly impacts therapeutic outcomes. Emerging research underscores the significance of considering the TME as a novel therapeutic strategy [16], aimed at dismantling the supportive network facilitating tumor growth and dissemination. This involves not only directly targeting tumor cells but also modulating the TME to restore normal homeostasis [17] and immune function, thereby enhancing the efficacy of both traditional and novel therapies (Fig. 2).

## Interactions between tumors and their microenvironment

Recent advances in oncologic therapeutics have precipitated a paradigmatic shift in the conceptualization of tumors. No longer seen as mere aggregates of cancerous cells, tumors are now understood as complex systems that are intricately intertwined with, and significantly influenced by, their surrounding inflammatory microenvironments [18, 19]. This recognition highlights the dual nature of tumors, comprising both the cancer cells themselves and the multifaceted inflammatory environment in which they exist and interact [20–22]. At the core of these complex interactions, a dynamic and heterogeneous entity comprising numerous cellular components [23–26], including immune cells, stromal cells, endothelial cells, and fibroblasts, along with non-cellular components such as the ECM, cytokines, chemokines, and various growth factors [25, 27–30]. The TME plays a crucial role in tumor progression, angiogenesis, invasion, and evasion of immune responses [31, 32]. The characteristic communication between tumor cells and their microenvironment is bidirectional [33], wherein tumor cells can reprogram the TME to support their own growth and survival, while components of the TME can influence the genetic and epigenetic landscape of tumor cells, thereby facilitating the evolution of cancer heterogeneity and drug resistance [34–36]. Inflammatory cells within the TME, such as tumor-associated macrophages (TAMs), myeloid-derived suppressor cells (MDSCs), and regulatory T cells (Tregs), often adopt a pro-tumorigenic phenotype, supporting tumor growth and suppressing anti-tumor immunity [10, 37]. Given the critical role of the TME in cancer biology, interest in targeting the TME as a therapeutic strategy keeps increasing. Approaches include modulating the immune microenvironment to enhance immunogenicity and immune responses to tumors, disrupting supportive stromal networks to inhibit tumor growth and metastasis, and targeting metabolic interactions between tumor cells and the TME to starve tumors of essential nutrients [38, 39]. In summary, the complex interactions between tumors and their microenvironments are central principles in cancer pathophysiology. Understanding this dynamic relationship is crucial for developing new therapeutic strategies that not only target tumor cells but also the complex ecosystem supporting their malignancy. Future research should focus on further elucidating the mechanisms of tumor–microenvironment interactions and leveraging this knowledge to overcome therapeutic resistance and improve clinical outcomes for cancer patients.

## The role of intratumoral microbiome—microenvironment in tumor development

As observed in cases such as breast and colon cancer, the transition from normal cells to clinically detectable tumor cells can span decades, mainly found in an inflammatory and pro-inflammatory microenvironments [40]. While genetic mutations remain the cornerstone of tumor-cell evolution, inflammatory mediators can act as catalysts for these genetic changes [41].

Pro-inflammatory carcinogens, including reactive oxygen species (ROS) and matrix metalloproteinases, can cause DNA damage and ECM degradation, respectively. In addition, certain cytokines, such as IL-1β, IL-6, IL-23, and tumor necrosis factor-α, can promote the proliferation of abnormal or precancerous cells, thereby facilitating tumor progression [42]. Chronic inflammation, in the presence of harmful stimuli, can both induce carcinogenic mutations and amplify genetic instability, further accelerating the carcinogenic process. The complex link between the inflammatory microenvironment and tumor development underscores a multifaceted process where genetic mutations and inflammatory mediators catalyze the transformation of normal cells into tumor entities over an extended period. This evolution, as seen in breast and colon cancers, is significantly influenced by a pro-inflammatory environment. Genetic alterations lay the foundational framework for tumor-cell evolution; however, it is the inflammatory mediators that expedite these mutations, acting as catalysts that not only accelerate genetic aberrations but also facilitate the progression of these cells toward malignancy.

**Fig. 3:**
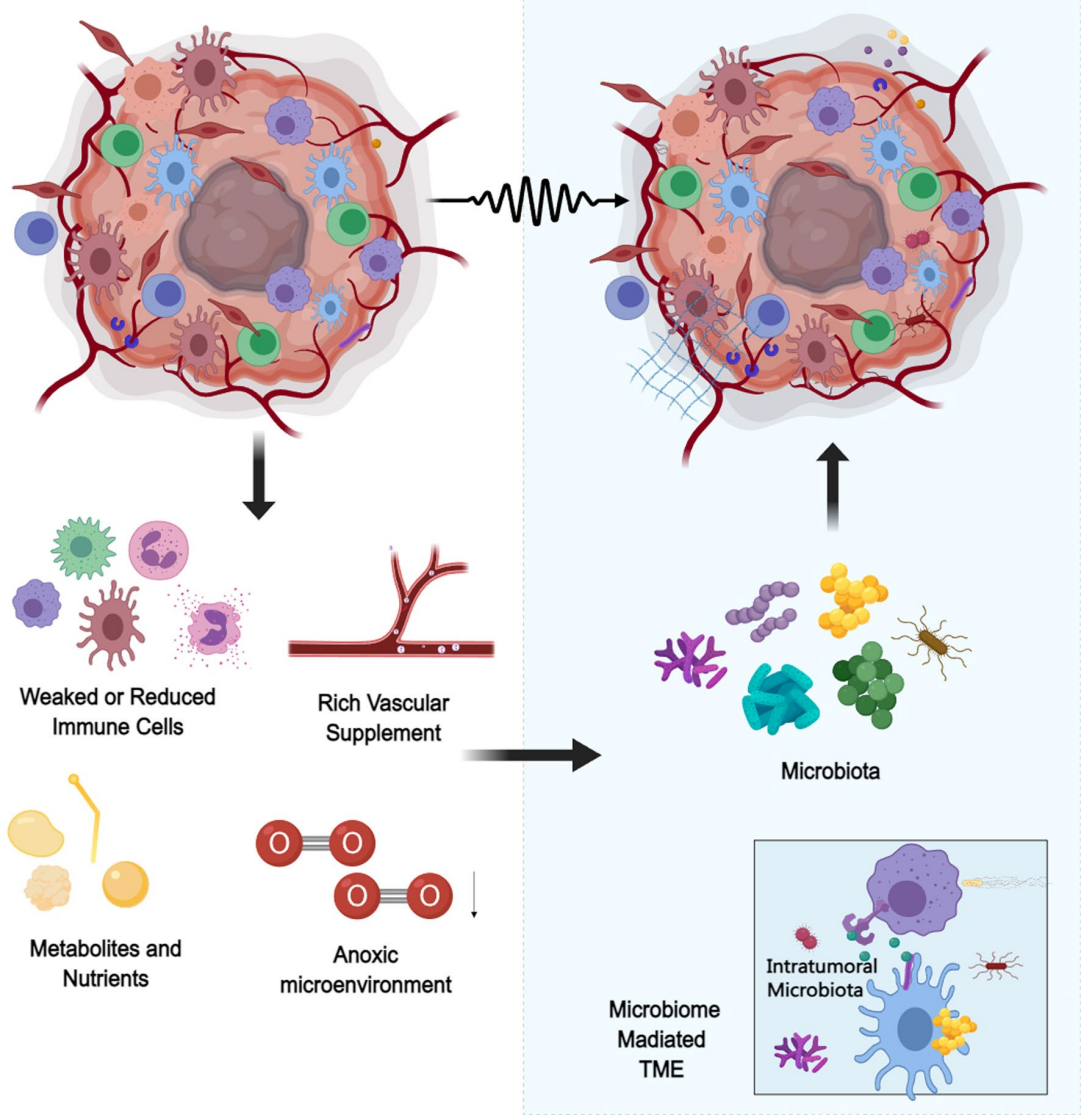
Aggregation and mechanism of action of microbiota in the tumor microenvironment

Recent studies have illuminated the multifaceted role of the TME, not only as a conducive milieu for tumor-cell growth and survival but also as an ecological niche for microbiota [43]. The TME, characterized by abundant nutrient supply, blood flow, and an immunosuppressive microenvironment, facilitates the migration and colonization of microorganisms. Multiple lines of evidence underscore the presence of microbiota within tumors, closely associated with cancer pathogenesis [44–47]. Tumor presence disrupts normal anatomic structures, providing opportunities for microbiota to migrate from adjacent organs to the TME. Consequently, microbiota integrate into and coexist with tumor cells within the TME. As integral components of the TME, tumor microbiota participate in cancer pathophysiology by enhancing oncogenic signals, modulating tumor metabolism, fostering an immunosuppressive microenvironment, and inducing chronic inflammation through various pathways, including enzymatic activity, toxin release, and metabolite generation [45, 48–50]. These microbial influences collectively promote tumor growth and metastasis, underscoring the intricate interplay between the tumor and its microbial inhabitants within the TME (Fig. 3).

The immunosuppressive environment in the TME weakens or reduces the activity of immune cells, making it easier for intratumoral microbiota to colonize. The tumor-associated vasculature is abundant but has reduced blood flow, providing a rich supply of nutrients for the intratumoral microbiota. The immunosuppressive environment in the TME weakens or reduces immune cell activity, making it easier for intratumoral microbiota to colonize. The tumor-associated vasculature is abundant but has reduced blood flow, providing a rich supply of nutrients for intratumoral microbiota. The TME is rich in metabolites and nutrients, creating a favorable environment for microbial growth. The anoxic state in the TME further promotes the survival and proliferation of intratumoral microbiota. These conditions lead to the establishment and aggregation of a unique microbiota (intratumoral microbiota) within the TME. Intratumoral microbiota further alter the TME through their metabolites and toxins, making it more suitable for tumor growth and metastasis.

The intratumoral microbiota wield significant influence over cancer occurrence and progression through multifaceted mechanisms. They prominently participate in tumorigenesis by instigating gene mutations and regulating metabolic processes. Bacterial gene toxins and metabolites inflict damage upon the DNA of target cells, thus instigating gene mutations [48]. For example, within *Escherichia coli* (*E. coli*), the pathogenic island housing polyketone synthase is known to trigger DNA double-strand breaks and interfere with the cell cycle [51]. Moreover, toxins secreted by *Pseudomonas aeruginosa* (*P. aeruginosa*) escalate ROS levels, precipitating DNA damage [52]. In addition, bacterial lipopolysaccharides (LPS) contribute to ROS elevation by perturbing mitochondrial metabolism, resulting in DNA base pair breaks, lipid peroxidation, and chromatin cross-linking [53]. Similarly, in pancreatic ductal adenocarcinoma (PDAC), the peptide arginine deaminase secreted by *Porphyromonas gingivalis* (*P. gingivalis*) escalates the mutation rate of TP53 and KRAS [54]. In a study by Liu et al., 14 intratumoral microbiota were identified as significantly associated with KRAS mutations and microsatellite instability through 16S rRNA sequencing [55].

Moreover, intratumoral microbiota influence cancer development by modulating oncogenic signaling pathways. For instance, *Streptococcus mitis* (*S. mitis*) upregulates the ERK and PI3K signaling pathways in lung cancer (LC), thus fostering cancer-cell proliferation [56]. Furthermore, bacterial LPS activate Toll-like receptor (TLR) 4, triggering the upregulation of inflammatory gene expression and facilitating tumorigenic proliferation [57]. Research indicates that *Fusobacterium nucleatum* (*F. nucleatum*) activates the autophagy pathway in colorectal cancer (CRC) cells via the TLR4 and related (TLR4/Keap1/NRF2, TLR4/NF-κB/S100A9, TLR4/ROS, NOD1/2, TLR4/P-PAK1) signaling pathways [58–61]. In addition, bacterial metabolites interact with G protein-coupled receptors, thereby influencing the activity of multiple pro-cancer or anti-cancer signaling pathways [62].

Inflammation and immune regulation also play pivotal roles in microbiota-mediated cancer progression. Microbial-induced chronic inflammation in tumors stimulates angiogenesis, thereby supplying nutrients to tumor cells and fostering tumor growth [63, 64]. Moreover, bacterial toxins and metabolites can induce the aggregation of inflammatory cells. In addition, the microbiota directly shape the local immune microenvironment. For example, *F. nucleatum* recruits tumor-associated macrophages and bone marrow-derived suppressor cells, fostering an immunosuppressive microenvironment [65]. The administration of antibiotics can eradicate intratumoral bacteria, enhancing the activity of M1 macrophages and promoting the growth and maturation of CD4+ and CD8+ T cells [66–68]. Furthermore, bacteria can stimulate the expansion of γδ T cells, leading to the release of IL-17 and IL-22, thereby promoting tumor progression [69]. Alam et al. discovered that fungi expedite cancer-cell proliferation by activating the CARD9 pathway, fostering IL-33 secretion, and increasing the infiltration of Th2 and lymphocytes [70].

Overall, the interplay between cancer microbiota, oncogenic signaling, inflammation, and immune regulation shapes tumor microenvironments and influences multiple biologic processes of tumors (Fig. 3).

## TME in cancer progression and metastasis

Metastatic tumors pose the greatest threat to the survival of cancer patients [27]. The pre-metastatic process involves primary tumors secreting factors that prime distant organs, creating a pre-metastatic niche conducive to tumor-cell colonization and proliferation [71]. Angiogenesis within the TME is pivotal for cancer-cell expansion and metastasis [72, 73]. As these vessels mature, they stimulate latent tumor cells to transform into aggressive entities.

The pre-metastatic niche is established by the secretion of various factors from primary tumors, which prepare distant organs for colonization of circulating tumor cells (CTCs) [27]. This niche provides a supportive environment for the proliferation of metastatic tumor cells. Crucially, angiogenesis within the TME is essential for tumor growth and metastasis [74]. Newly formed blood vessels supply necessary nutrients and oxygen, creating a sanctuary for dormant tumor cells within the bone marrow. As these vessels mature, they activate these dormant cells, converting them into aggressive, metastatic tumors [73]. This process is facilitated by the interaction of cancer cells with the surrounding stromal and immune cells, which remodel the ECM and promote angiogenesis.

**Fig. 4:**
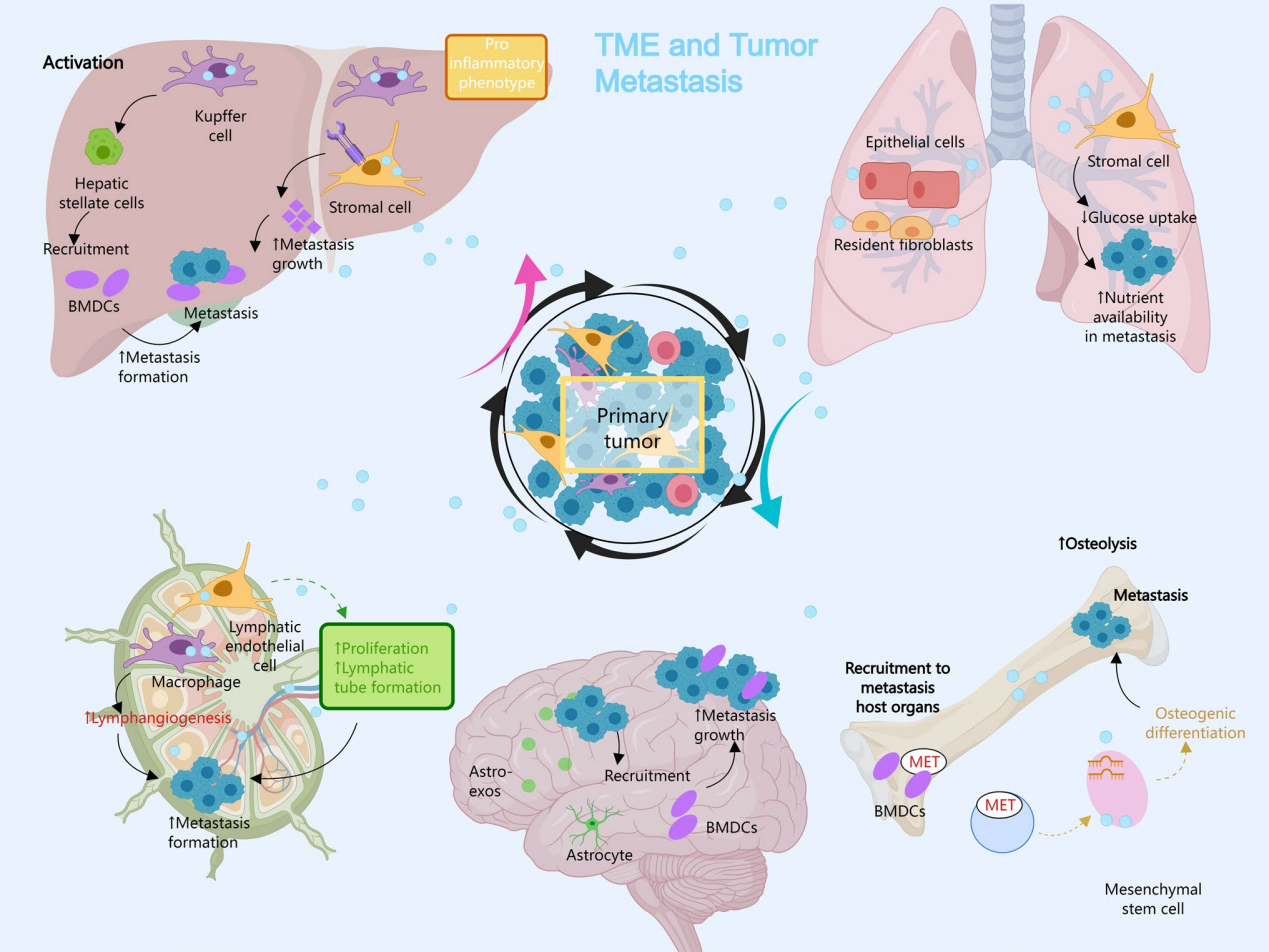
The TME influences the metastatic spread of tumors to various organs. The primary tumor manipulates the surrounding microenvironment by recruiting immune and stromal cells, creating a pro-tumorigenic niche that supports tumor growth and metastasis. Kupffer cells and hepatic stellate cells are activated, adopting a pro-inflammatory phenotype and recruiting bone marrow-derived cells (BMDCs). Activated stromal cells promote metastasis growth in the liver. In the lung, stromal and epithelial cells facilitate metastasis through increased glucose uptake and nutrient availability. Macrophages stimulate lymphangiogenesis by promoting lymphatic endothelial cell proliferation and lymphatic tube formation, aiding in metastasis formation. Astrocytes release exosomes (Astro-exos) that recruit BMDCs and facilitate metastasis growth in the brain. Metastatic tumor cells cause osteolysis, leading to bone metastasis formation. Mesenchymal stem cells (MSCs) undergo osteogenic differentiation, contributing to the bone metastatic niche. BMDCs are recruited to metastatic host organs

Furthermore, the destiny of metastatic cells within the bloodstream is determined by various immune cell types [27]. Platelets and neutrophils support the survival of CTCs by protecting them from physical stress and immune attacks, whereas NK cells and other adaptive immune cells can eliminate CTCs. The co-option of immune cells, tissue-resident cells, and cancer-associated fibroblasts (CAFs) by tumor cells fosters the invasive behavior of cancer, further enhancing their metastatic potential [75, 76] (Fig. 4).

## Discussion

The TME is instrumental in orchestrating tumor progression, metastasis, and response to therapy. It serves as a nurturing ground for tumor cells while actively undergoing remodeling by these cells to foster malignancy. This intricate symbiosis underscores the therapeutic potential of targeting the TME. Innovations in targeting the TME’s vascular architecture, leveraging immune checkpoint inhibitors, and deploying T-cell therapies have marked significant milestones in clinical success, underscoring the viability of these strategies in oncologic management. Nonetheless, the TME’s inherent complexity and heterogeneity pose substantial hurdles to attaining optimal therapeutic benefits. A profound comprehension of these interactions is paramount for the formulation of more efficacious therapeutic modalities.

To augment the effectiveness of therapies directed at the TME, it is imperative to amalgamate insights from the biologic intricacies within the TME with cutting-edge therapeutic modalities. This encompasses the development of pharmacologic agents with the precision to target distinct constituents of the TME, alongside strategies to modulate the TME toward reinstating normal tissue equilibrium and bolstering anti-tumor immune responses. For example, integrating TME-targeted pharmacotherapies with conventional chemotherapy or immunotherapy could surmount resistance mechanisms, thereby enhancing patient outcomes. Furthermore, identifying biomarkers predictive of responses to TME-targeted interventions is crucial for the personalization of oncologic therapy. By discerning patients poised to derive benefit from these therapies based on their TME characteristics, clinicians can customize therapeutic regimens tailored to individual patient profiles, thereby advancing the precision and efficacy of cancer treatment.

In conclusion, the TME stands as a pivotal frontier in oncologic research and therapy. Advancing our understanding of the complex interplay within the TME and innovating therapeutic strategies aimed at these dynamics promise to revolutionize cancer treatment. This endeavor necessitates a synergistic collaboration among researchers, clinical practitioners, and pharmaceutical innovators to convert scientific discoveries into therapeutic realities, ultimately ameliorating the prognosis and enhancing the quality of life for individuals afflicted with cancer.

## Data Availability

Not applicable.

## References

[1] Yoo HC, Han JM. Amino acid metabolism in cancer drug resistance. Cells. 2022;11(1):140. 10.3390/cells11010140. (**Published 2022 Jan 2**). 35011702 10.3390/cells11010140 PMC8750102

[2] Sun Y, Wang R, Qiao M, Xu Y, Guan W, Wang L. Cancer associated fibroblasts tailored tumor microenvironment of therapy resistance in gastrointestinal cancers. J Cell Physiol. 2018;233(9):6359–69. 10.1002/jcp.26433. 29334123 10.1002/jcp.26433 PMC13482468

[3] Sun Y. Tumor microenvironment and cancer therapy resistance. Cancer Lett. 2016;380(1):205–15. 10.1016/j.canlet.2015.07.044. 26272180 10.1016/j.canlet.2015.07.044

[4] Silva VL, Al-Jamal WT. Exploiting the cancer niche: tumor-associated macrophages and hypoxia as promising synergistic targets for nano-based therapy. J Control Release. 2017;253:82–96. 10.1016/j.jconrel.2017.03.013. 28285930 10.1016/j.jconrel.2017.03.013

[5] Kumari S, Advani D, Sharma S, Ambasta RK, Kumar P. Combinatorial therapy in tumor microenvironment: where do we stand? Biochim Biophys Acta Rev Cancer. 2021;1876(2): 188585. 10.1016/j.bbcan.2021.188585. 34224836 10.1016/j.bbcan.2021.188585

[6] Chen F, Zhuang X, Lin L, Yu P, Wang Y, Shi Y, et al. New horizons in tumor microenvironment biology: challenges and opportunities. BMC Med. 2015;13:45. 10.1186/s12916-015-0278-7. (**Published 2015 Mar 5**). 25857315 10.1186/s12916-015-0278-7 PMC4350882

[7] Zhou H, Wang M, Zhang Y, Su Q, Xie Z, Chen X, et al. Functions and clinical significance of mechanical tumor microenvironment: cancer cell sensing, mechanobiology and metastasis. Cancer Commun (Lond). 2022;42(5):374–400. 10.1002/cac2.12294. 35470988 10.1002/cac2.12294 PMC9118059

[8] Petitprez F, Sun CM, Lacroix L, Sautès-Fridman C, de Reyniès A, Fridman WH. Quantitative analyses of the tumor microenvironment composition and orientation in the era of precision medicine. Front Oncol. 2018;8:390. 10.3389/fonc.2018.00390. (**Published 2018 Sep 25**). 30319963 10.3389/fonc.2018.00390 PMC6167550

[9] Ma K, Zhang L. Overview: lipid metabolism in the tumor microenvironment. Adv Exp Med Biol. 2021;1316:41–7. 10.1007/978-981-33-6785-2_3. 33740242 10.1007/978-981-33-6785-2_3

[10] Anderson NM, Simon MC. The tumor microenvironment. Curr Biol. 2020;30(16):R921–5. 10.1016/j.cub.2020.06.081. 32810447 10.1016/j.cub.2020.06.081 PMC8194051

[11] Abadjian MZ, Edwards WB, Anderson CJ. Imaging the tumor microenvironment. Adv Exp Med Biol. 2017;1036:229–57. 10.1007/978-3-319-67577-0_15. 29275475 10.1007/978-3-319-67577-0_15

[12] Fowler NH, Cheah CY, Gascoyne RD, Gribben J, Neelapu SS, Ghia P, et al. Role of the tumor microenvironment in mature B-cell lymphoid malignancies. Haematologica. 2016;101(5):531–40. 10.3324/haematol.2015.139493. 27132279 10.3324/haematol.2015.139493 PMC5004369

[13] Kao KC, Vilbois S, Tsai CH, Ho PC. Metabolic communication in the tumour-immune microenvironment. Nat Cell Biol. 2022;24(11):1574–83. 10.1038/s41556-022-01002-x. 36229606 10.1038/s41556-022-01002-x

[14] Farc O, Cristea V. An overview of the tumor microenvironment, from cells to complex networks (review). Exp Ther Med. 2021;21(1):96. 10.3892/etm.2020.9528. 33363607 10.3892/etm.2020.9528 PMC7725019

[15] Khalaf K, Hana D, Chou JT, Singh C, Mackiewicz A, Kaczmarek M. Aspects of the tumor microenvironment involved in immune resistance and drug resistance. Front Immunol. 2021;12:656364. 10.3389/fimmu.2021.656364. (**Published 2021 May 27**). 34122412 10.3389/fimmu.2021.656364 PMC8190405

[16] Maman S, Witz IP. A history of exploring cancer in context. Nat Rev Cancer. 2018;18(6):359–76. 10.1038/s41568-018-0006-7. 29700396 10.1038/s41568-018-0006-7

[17] Suwa T, Kobayashi M, Nam JM, Harada H. Tumor microenvironment and radioresistance. Exp Mol Med. 2021;53(6):1029–35. 10.1038/s12276-021-00640-9. 34135469 10.1038/s12276-021-00640-9 PMC8257724

[18] Cheng YQ, Wang SB, Liu JH, Jin L, Liu Y, Li CY, et al. Modifying the tumour microenvironment and reverting tumour cells: new strategies for treating malignant tumours. Cell Prolif. 2020;53(8): e12865. 10.1111/cpr.12865. 32588948 10.1111/cpr.12865 PMC7445401

[19] Bejarano L, Jordāo MJC, Joyce JA. Therapeutic targeting of the tumor microenvironment. Cancer Discov. 2021;11(4):933–59. 10.1158/2159-8290.CD-20-1808. 33811125 10.1158/2159-8290.CD-20-1808

[20] Denk D, Greten FR. Inflammation: the incubator of the tumor microenvironment. Trends Cancer. 2022;8(11):901–14. 10.1016/j.trecan.2022.07.002. 35907753 10.1016/j.trecan.2022.07.002

[21] Yuan Y, Li H, Pu W, Chen L, Guo D, Jiang H, et al. Cancer metabolism and tumor microenvironment: fostering each other? Sci China Life Sci. 2022;65(2):236–79. 10.1007/s11427-021-1999-2. 34846643 10.1007/s11427-021-1999-2

[22] Kennel KB, Bozlar M, De Valk AF, Greten FR. Cancer-associated fibroblasts in inflammation and antitumor immunity. Clin Cancer Res. 2023;29(6):1009–16. 10.1158/1078-0432.CCR-22-1031. 36399325 10.1158/1078-0432.CCR-22-1031 PMC10011884

[23] Schulz M, Salamero-Boix A, Niesel K, Alekseeva T, Sevenich L. Microenvironmental regulation of tumor progression and therapeutic response in brain metastasis. Front Immunol. 2019;10:1713. 10.3389/fimmu.2019.01713. (**Published 2019 Jul 24.**). 31396225 10.3389/fimmu.2019.01713 PMC6667643

[24] Yin Z, Bai L, Li W, Zeng T, Tian H, Cui J. Targeting T cell metabolism in the tumor microenvironment: an anti-cancer therapeutic strategy. J Exp Clin Cancer Res. 2019;38(1):403. 10.1186/s13046-019-1409-3. (**Published 2019 Sep 13**). 31519198 10.1186/s13046-019-1409-3 PMC6743108

[25] Bayik D, Lathia JD. Cancer stem cell-immune cell crosstalk in tumour progression. Nat Rev Cancer. 2021;21(8):526–36. 10.1038/s41568-021-00366-w. 34103704 10.1038/s41568-021-00366-w PMC8740903

[26] Chen D, Zhang X, Li Z, Zhu B. Metabolic regulatory crosstalk between tumor microenvironment and tumor-associated macrophages. Theranostics. 2021;11(3):1016–30. 10.7150/thno.51777. (**Published 2021 Jan 1**). 33391518 10.7150/thno.51777 PMC7738889

[27] de Visser KE, Joyce JA. The evolving tumor microenvironment: From cancer initiation to metastatic outgrowth. Cancer Cell. 2023;41(3):374–403. 10.1016/j.ccell.2023.02.016. 36917948 10.1016/j.ccell.2023.02.016

[28] Vitale I, Manic G, Coussens LM, Kroemer G, Galluzzi L. Macrophages and metabolism in the tumor microenvironment. Cell Metab. 2019;30(1):36–50. 10.1016/j.cmet.2019.06.001. 31269428 10.1016/j.cmet.2019.06.001

[29] Downs-Canner SM, Meier J, Vincent BG, Serody JS. B cell function in the tumor microenvironment. Annu Rev Immunol. 2022;40:169–93. 10.1146/annurev-immunol-101220-015603. 35044794 10.1146/annurev-immunol-101220-015603

[30] Dey P, Kimmelman AC, DePinho RA. Metabolic codependencies in the tumor microenvironment. Cancer Discov. 2021;11(5):1067–81. 10.1158/2159-8290.CD-20-1211. 33504580 10.1158/2159-8290.CD-20-1211 PMC8102306

[31] Tiwari A, Trivedi R, Lin SY. Tumor microenvironment: barrier or opportunity towards effective cancer therapy. J Biomed Sci. 2022;29(1):83. 10.1186/s12929-022-00866-3. (**Published 2022 Oct 17**). 36253762 10.1186/s12929-022-00866-3 PMC9575280

[32] Hinshaw DC, Shevde LA. The tumor microenvironment innately modulates cancer progression. Cancer Res. 2019;79(18):4557–66. 10.1158/0008-5472.CAN-18-3962. 31350295 10.1158/0008-5472.CAN-18-3962 PMC6744958

[33] Xiao Y, Yu D. Tumor microenvironment as a therapeutic target in cancer. Pharmacol Ther. 2021;221: 107753. 10.1016/j.pharmthera.2020.107753. 33259885 10.1016/j.pharmthera.2020.107753 PMC8084948

[34] Zhang A, Miao K, Sun H, Deng CX. Tumor heterogeneity reshapes the tumor microenvironment to influence drug resistance. Int J Biol Sci. 2022;18(7):3019–33. 10.7150/ijbs.72534. (**Published 2022 Apr 24**). 35541919 10.7150/ijbs.72534 PMC9066118

[35] Arner EN, Rathmell JC. Metabolic programming and immune suppression in the tumor microenvironment. Cancer Cell. 2023;41(3):421–33. 10.1016/j.ccell.2023.01.009. 36801000 10.1016/j.ccell.2023.01.009 PMC10023409

[36] Nussinov R, Tsai CJ, Jang H. Anticancer drug resistance: an update and perspective. Drug Resist Updat. 2021;59: 100796. 10.1016/j.drup.2021.100796. 34953682 10.1016/j.drup.2021.100796 PMC8810687

[37] Liu Q, Luo Q, Ju Y, Song G. Role of the mechanical microenvironment in cancer development and progression. Cancer Biol Med. 2020;17(2):282–92. 10.20892/j.issn.2095-3941.2019.0437. 32587769 10.20892/j.issn.2095-3941.2019.0437 PMC7309462

[38] Thakkar S, Sharma D, Kalia K, Tekade RK. Tumor microenvironment targeted nanotherapeutics for cancer therapy and diagnosis: a review. Acta Biomater. 2020;101:43–68. 10.1016/j.actbio.2019.09.009. 31518706 10.1016/j.actbio.2019.09.009

[39] Sounni NE, Noel A. Targeting the tumor microenvironment for cancer therapy. Clin Chem. 2013;59(1):85–93. 10.1373/clinchem.2012.185363. 23193058 10.1373/clinchem.2012.185363

[40] Shiao SL, Ganesan AP, Rugo HS, Coussens LM. Immune microenvironments in solid tumors: new targets for therapy. Genes Dev. 2011;25(24):2559–72. 10.1101/gad.169029.111. 22190457 10.1101/gad.169029.111 PMC3248678

[41] Mempel TR, Lill JK, Altenburger LM. How chemokines organize the tumour microenvironment. Nat Rev Cancer. 2024;24(1):28–50. 10.1038/s41568-023-00635-w. 38066335 10.1038/s41568-023-00635-w PMC11480775

[42] Ozga AJ, Chow MT, Luster AD. Chemokines and the immune response to cancer. Immunity. 2021;54(5):859–74. 10.1016/j.immuni.2021.01.012. 33838745 10.1016/j.immuni.2021.01.012 PMC8434759

[43] Wong-Rolle A, Wei HK, Zhao C, Jin C. Unexpected guests in the tumor microenvironment: microbiome in cancer. Protein Cell. 2021;12(5):426–35. 10.1007/s13238-020-00813-8. 33296049 10.1007/s13238-020-00813-8 PMC8106554

[44] Yang Q, Wang B, Zheng Q, Li H, Meng X, Zhou F, et al. A review of gut microbiota-derived metabolites in tumor progression and cancer therapy. Adv Sci (Weinh). 2023;10(15): e2207366. 10.1002/advs.202207366. 36951547 10.1002/advs.202207366 PMC10214247

[45] Sepich-Poore GD, Zitvogel L, Straussman R, Hasty J, Wargo JA, Knight R. The microbiome and human cancer. Science. 2021;371(6536):eabc4552. 10.1126/science.abc4552. 33766858 10.1126/science.abc4552 PMC8767999

[46] Xie Y, Xie F, Zhou X, Zhang L, Yang B, Huang J, et al. Microbiota in tumors: from understanding to application. Adv Sci (Weinh). 2022;9(21): e2200470. 10.1002/advs.202200470. 35603968 10.1002/advs.202200470 PMC9313476

[47] El Tekle G, Garrett WS. Bacteria in cancer initiation, promotion and progression. Nat Rev Cancer. 2023;23(9):600–18. 10.1038/s41568-023-00594-2. 37400581 10.1038/s41568-023-00594-2

[48] Yang L, Li A, Wang Y, Zhang Y. Intratumoral microbiota: roles in cancer initiation, development and therapeutic efficacy. Signal Transduct Target Ther. 2023;8(1):35. 10.1038/s41392-022-01304-4. (**Published 2023 Jan 16**). 36646684 10.1038/s41392-022-01304-4 PMC9842669

[49] Fu A, Yao B, Dong T, Cai S. Emerging roles of intratumor microbiota in cancer metastasis. Trends Cell Biol. 2023;33(7):583–93. 10.1016/j.tcb.2022.11.007. 36522234 10.1016/j.tcb.2022.11.007

[50] Ferrari V, Rescigno M. The intratumoral microbiota: friend or foe? Trends Cancer. 2023;9(6):472–9. 10.1016/j.trecan.2023.03.005. 37061408 10.1016/j.trecan.2023.03.005

[51] Wu J, Zhang P, Mei W, Zeng C. Intratumoral microbiota: implications for cancer onset, progression, and therapy. Front Immunol. 2024;14:1301506. 10.3389/fimmu.2023.1301506. (**Published 2024 Jan 16**). 38292482 10.3389/fimmu.2023.1301506 PMC10824977

[52] Yang J, Wang Q, Wang C, Yang R, Ahmed M, Kumaran S, et al. Pseudomonas aeruginosa synthesized silver nanoparticles inhibit cell proliferation and induce ROS mediated apoptosis in thyroid cancer cell line (TPC1). Artif Cells Nanomed Biotechnol. 2020;48(1):800–9. 10.1080/21691401.2019.1687495. 32432484 10.1080/21691401.2019.1687495

[53] Cui J, Chen Y, Wang HY, Wang RF. Mechanisms and pathways of innate immune activation and regulation in health and cancer. Hum Vaccin Immunother. 2014;10(11):3270–85. 10.4161/21645515.2014.979640. 25625930 10.4161/21645515.2014.979640 PMC4514086

[54] Saba E, Farhat M, Daoud A, Khashan A, Forkush E, Menahem NH, et al. Oral bacteria accelerate pancreatic cancer development in mice. Gut. 2024;73(5):770–86. 10.1136/gutjnl-2023-330941. (**Published 2024 Apr 5**). 38233197 10.1136/gutjnl-2023-330941

[55] Liu W, Zhang X, Xu H, Li S, Lau HC, Chen Q, et al. Microbial community heterogeneity within colorectal neoplasia and its correlation with colorectal carcinogenesis. Gastroenterology. 2021;160(7):2395–408. 10.1053/j.gastro.2021.02.020. 33581124 10.1053/j.gastro.2021.02.020

[56] Tsay JJ, Wu BG, Badri MH, Clemente JC, Shen N, Meyn P, et al. Airway microbiota is associated with upregulation of the PI3K pathway in lung cancer. Am J Respir Crit Care Med. 2018;198(9):1188–98. 10.1164/rccm.201710-2118OC. 29864375 10.1164/rccm.201710-2118OC PMC6221574

[57] Rezania S, Amirmozaffari N, Rashidi N, et al. The same and not the same: heterogeneous functional activation of prostate tumor cells by TLR ligation. Cancer Cell Int. 2014;14:54. 10.1186/1475-2867-14-54. (**Published 2014 Jun 19**). 24966802 10.1186/1475-2867-14-54 PMC4069277

[58] Kong C, Yan X, Zhu Y, Zhu H, Luo Y, Liu P, et al. Fusobacterium nucleatum promotes the development of colorectal cancer by activating a cytochrome P450/epoxyoctadecenoic acid axis via TLR4/Keap1/NRF2 signaling. Cancer Res. 2021;81(17):4485–98. 10.1158/0008-5472.CAN-21-0453. 34162680 10.1158/0008-5472.CAN-21-0453

[59] Kong X, Zhang Y, Xiang L, You Y, Duan Y, Zhao Y, et al. Fusobacterium nucleatum-triggered neutrophil extracellular traps facilitate colorectal carcinoma progression. J Exp Clin Cancer Res. 2023;42(1):236. 10.1186/s13046-023-02817-8. (**Published 2023 Sep 9**). 37684625 10.1186/s13046-023-02817-8 PMC10492297

[60] Chen Y, Peng Y, Yu J, Chen T, Wu Y, Shi L, et al. Invasive Fusobacterium nucleatum activates beta-catenin signaling in colorectal cancer via a TLR4/P-PAK1 cascade. Oncotarget. 2017;8(19):31802–14. 10.18632/oncotarget.15992. 28423670 10.18632/oncotarget.15992 PMC5458249

[61] Li S, Liu J, Zheng X, Ren L, Yang Y, Li W, et al. Tumorigenic bacteria in colorectal cancer: mechanisms and treatments. Cancer Biol Med. 2021. 10.20892/j.issn.2095-3941.2020.0651. (**Published online September 30, 2021**). 34586760 10.20892/j.issn.2095-3941.2020.0651 PMC8832957

[62] van Senten JR, Fan TS, Siderius M, Smit MJ. Viral G protein-coupled receptors as modulators of cancer hallmarks. Pharmacol Res. 2020;156: 104804. 10.1016/j.phrs.2020.104804. 32278040 10.1016/j.phrs.2020.104804

[63] Mortara L, Benest AV, Derosa L, Chouaib S, Ribatti D. Editorial: The intricate innate immune-cancer cell relationship in the context of tumor angiogenesis, immunity and microbiota: the angiogenic switch in the tumor microenvironment as a key target for immunotherapies. Front Immunol. 2022;13:1045074. 10.3389/fimmu.2022.1045074. (**Published 2022 Oct 6**). 36275734 10.3389/fimmu.2022.1045074 PMC9583657

[64] Laplane L, Duluc D, Bikfalvi A, Larmonier N, Pradeu T. Beyond the tumour microenvironment [published correction appears in Int J Cancer. 2021 Mar 15;148(6):E5. 10.1002/ijc.33412]. Int J Cancer. 2019;145(10):2611–8. 10.1002/ijc.32343. 30989643 10.1002/ijc.32343 PMC6766895

[65] Xu C, Fan L, Lin Y, Shen W, Qi Y, Zhang Y, et al. Fusobacterium nucleatum promotes colorectal cancer metastasis through miR-1322/CCL20 axis and M2 polarization. Gut Microbes. 2021;13(1):1980347. 10.1080/19490976.2021.1980347. 34632963 10.1080/19490976.2021.1980347 PMC8510564

[66] Uribe-Herranz M, Bittinger K, Rafail S, Guedan S, Pierini S, Tanes C, et al. Gut microbiota modulates adoptive cell therapy via CD8α dendritic cells and IL-12. JCI Insight. 2018;3(4):e94952. 10.1172/jci.insight.94952. (**Published 2018 Feb 22**). 29467322 10.1172/jci.insight.94952 PMC5916241

[67] Uribe-Herranz M, Rafail S, Beghi S, Gil-de-Gómez L, Verginadis I, Bittinger K, et al. Gut microbiota modulate dendritic cell antigen presentation and radiotherapy-induced antitumor immune response. J Clin Investig. 2020;130(1):466–79. 10.1172/JCI124332. 31815742 10.1172/JCI124332 PMC6934221

[68] Wang Y, Han Y, Yang C, Bai T, Zhang C, Wang Z, et al. Long-term relapse-free survival enabled by integrating targeted antibacteria in antitumor treatment. Nat Commun. 2024;15(1):4194. 10.1038/s41467-024-48662-x. (**Published 2024 May 17**). 38760364 10.1038/s41467-024-48662-x PMC11101653

[69] Ma J, Huang L, Hu D, Zeng S, Han Y, Shen H. The role of the tumor microbe microenvironment in the tumor immune microenvironment: bystander, activator, or inhibitor? J Exp Clin Cancer Res. 2021;40(1):327. 10.1186/s13046-021-02128-w. (**Published 2021 Oct 16**). 34656142 10.1186/s13046-021-02128-w PMC8520212

[70] Alam A, Levanduski E, Denz P, Villavicencio HS, Bhatta M, Alhorebi L, et al. Fungal mycobiome drives IL-33 secretion and type 2 immunity in pancreatic cancer. Cancer Cell. 2022;40(2):153-167.e11. 10.1016/j.ccell.2022.01.003. 35120601 10.1016/j.ccell.2022.01.003 PMC8847236

[71] Fares J, Fares MY, Khachfe HH, Salhab HA, Fares Y. Molecular principles of metastasis: a hallmark of cancer revisited. Signal Transduct Target Ther. 2020;5(1):28. 10.1038/s41392-020-0134-x. (**Published 2020 Mar 12**). 32296047 10.1038/s41392-020-0134-x PMC7067809

[72] Pitt JM, Marabelle A, Eggermont A, Soria JC, Kroemer G, Zitvogel L. Targeting the tumor microenvironment: removing obstruction to anticancer immune responses and immunotherapy. Ann Oncol. 2016;27(8):1482–92. 10.1093/annonc/mdw168. 27069014 10.1093/annonc/mdw168

[73] Jiang X, Wang J, Deng X, Xiong F, Zhang S, Gong Z, et al. The role of microenvironment in tumor angiogenesis. J Exp Clin Cancer Res. 2020;39(1):204. 10.1186/s13046-020-01709-5. (**Published 2020 Sep 30**). 32993787 10.1186/s13046-020-01709-5 PMC7526376

[74] Siddhartha R, Garg M. Interplay between extracellular matrix remodeling and angiogenesis in tumor ecosystem. Mol Cancer Ther. 2023;22(3):291–305. 10.1158/1535-7163.MCT-22-0595. 36861362 10.1158/1535-7163.MCT-22-0595

[75] Lambert AW, Zhang Y, Weinberg RA. Cell-intrinsic and microenvironmental determinants of metastatic colonization [published correction appears in Nat Cell Biol. 2024 Jul;26(7):1225. 10.1038/s41556-024-01458-z]. Nat Cell Biol. 2024;26(5):687–97. 10.1038/s41556-024-01409-8. 38714854 10.1038/s41556-024-01409-8

[76] Zhang H, Yue X, Chen Z, Liu C, Wu W, Zhang N, et al. Define cancer-associated fibroblasts (CAFs) in the tumor microenvironment: new opportunities in cancer immunotherapy and advances in clinical trials. Mol Cancer. 2023;22(1):159. 10.1186/s12943-023-01860-5. (**Published 2023 Oct 2**). 37784082 10.1186/s12943-023-01860-5 PMC10544417

